# Supplementary material for: Centre-of-mass and minimal speed limits of the great hammerhead
Source: R Soc Open Sci. 2020 Oct 14;7(10):200864. doi: 10.1098/rsos.200864 (PMC7657883; doi:10.1098/rsos.200864)
Supplement: Supplementary figures [file rsos200864supp1.pdf]

Center-of-mass and minimal speed limits of the great hammerhead.

Supplementary figures.

Gil Iosilevskii[1]

Faculty of Aerospace Engineering, Technion, Haifa, Israel

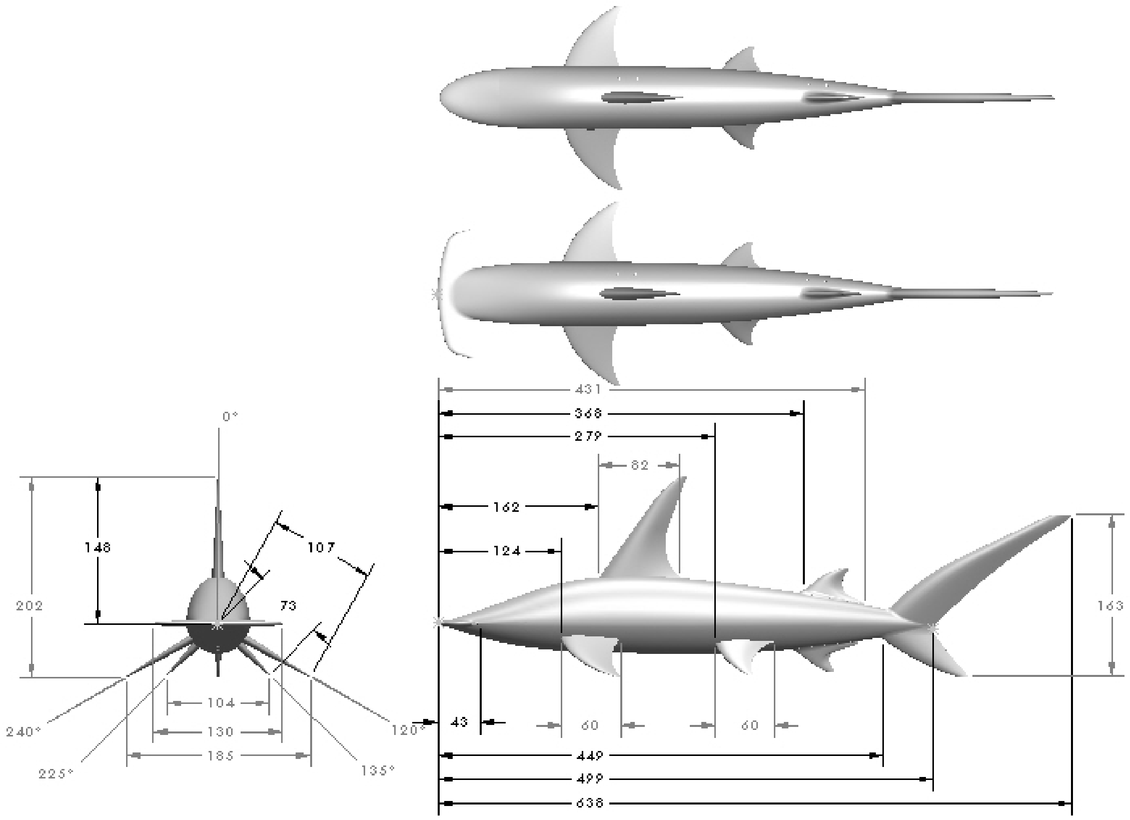

Figure S1: Multi-view projections of the model. Removing the cephalofoil leaves the cranial and lateral views (practically) unaltered; the altered dorsal view is shown on the top.

[1] igil@technion.ac.il

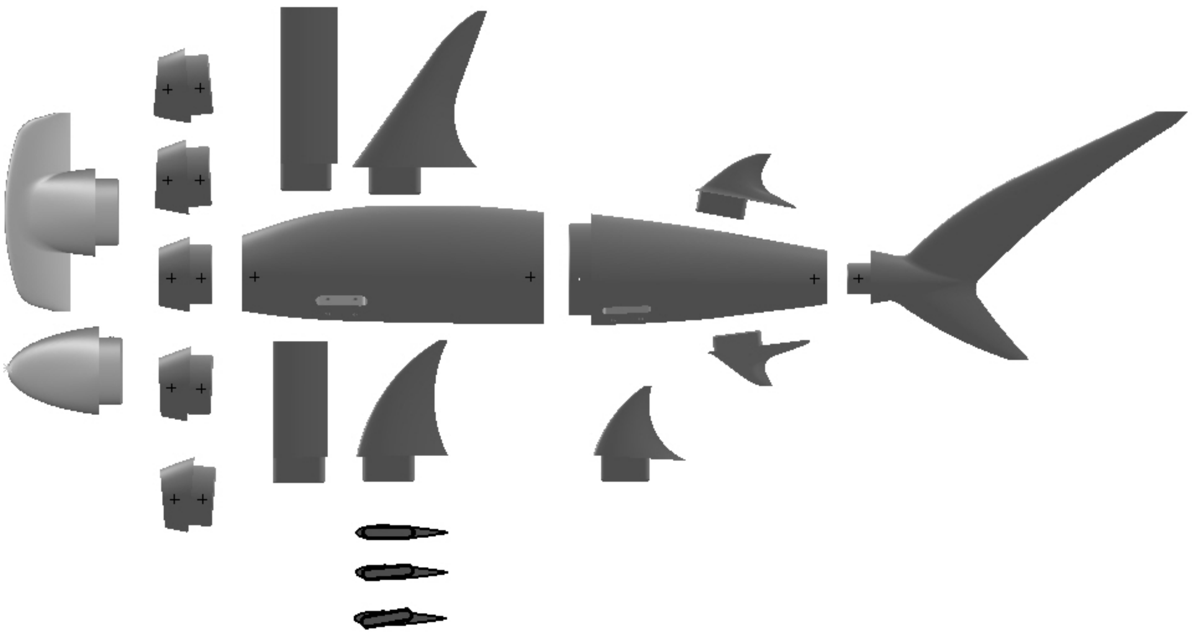

Figure S2: The pieces of the model: two heads (dorsal view), five necks (lateral view), two equivalent dorsal fins (crescent and rectangular), two sets of equivalent pectoral fins (crescent and rectangular), a pair of pelvic fins, the anal and the second dorsal fin, the caudal fin (it was never used), and two body pieces with attachment ports. Crescent pectoral fins were printed in five variants, differing in their anchor orientation (ventral view of three of them is shown).

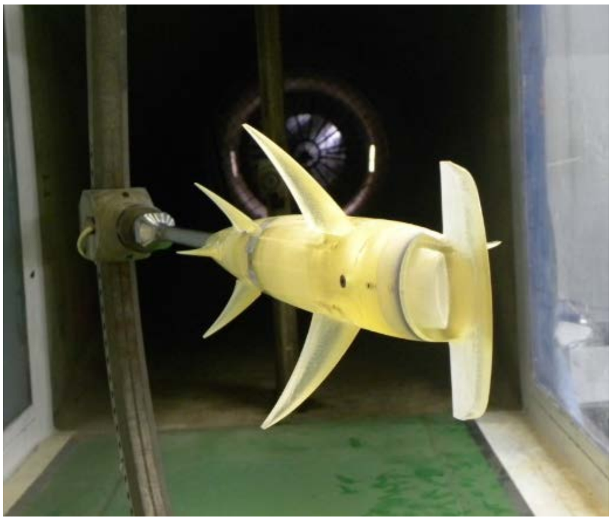

Figure S3: The model in the wind tunnel. Moving the model left-to-right simulates pitching up. The suction fan of the wind tunnel is seen in the rear. The cross section is 1m by 1m by 3m. The metal cylinder sticking from the caudal end is the sting of the 6 component balance.

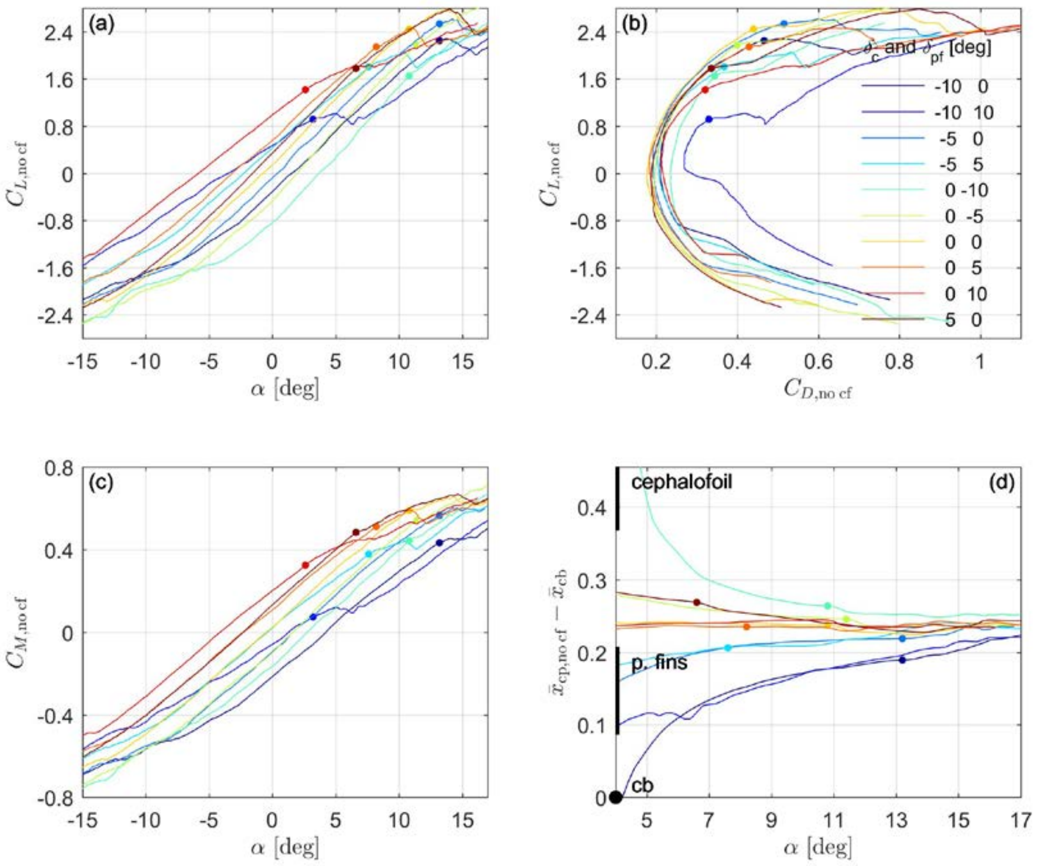

Figure S4: The lift coefficient (a), the pitching moment coefficient (c) and the center of pressure (d) as functions of the angle-of-attack (*α*) for ten different orientations of the cephalofoil and the pectoral fins relative to the body. The respective drag polars are shown on (b). Small dots mark an estimated onset of the dorsal-side stall of the cephalofoil or the pectoral fins (it is the cephalofoil when $\delta_c \geq 0$). All surfaces stall when set at 11° to 13° relative to the swimming direction far from the shark. Thick vertical lines on (d) mark the extent of the cephalofoil and pectoral fins along the body. 'cb' marks the center of buoyancy. $\bar{x} = x/l$.

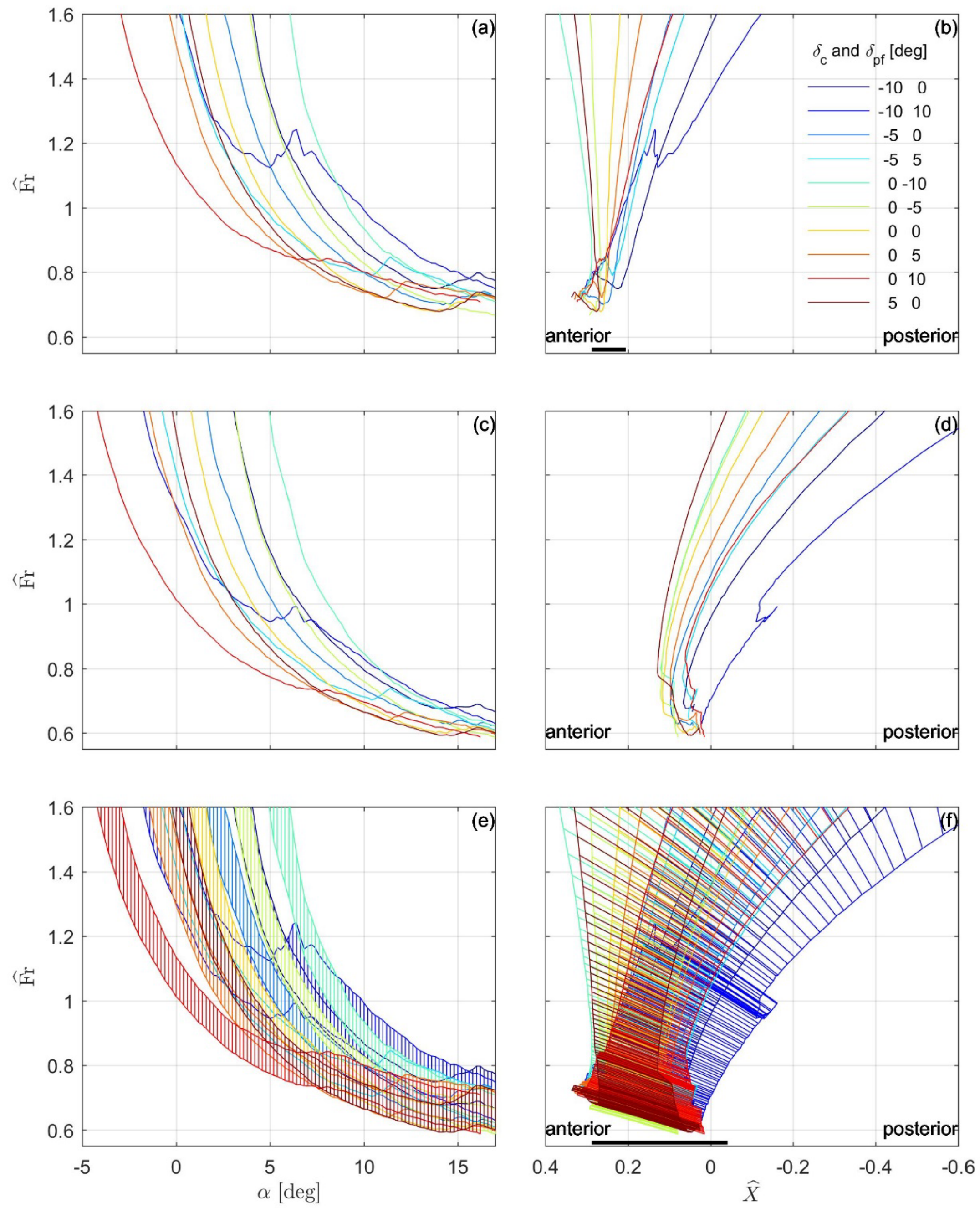

Figure S5: The scaled speed (a, c, e) and the scaled distance between the centers of mass and buoyancy (b, d, f) (positive when the center-of-mass is anteriad of the center-of-buoyancy) that balance the shark at $\gamma = 0$ with $\lambda_{cf} = 0$ (a, b), $\lambda_{cf} = 1$ (c, d), and $\lambda_{cf} \in (0,1)$ (e, f). Vertical lines on (e) and slanted lines on (f) connect the balance points with $\lambda_{cf} = 0$ to balance points with $\lambda_{cf} = 1$. Thick horizontal lines on (b) and (e) mark the range of center-of-mass positions that allow swimming at any $\widehat{\mathrm{Fr}} > 0.8$. There is no such a range with $\lambda_{cf} = 1$. For a 2.5 m shark with $\beta = 0.04$, $\widehat{\mathrm{Fr}}$ is numerically the same as the swim speed in m/s, whereas $\widehat{X}$ is numerically the same as the distance between the center-of-mass and the center-of-buoyancy in decimeters. This figure is based on the data shown on Fig. S4.

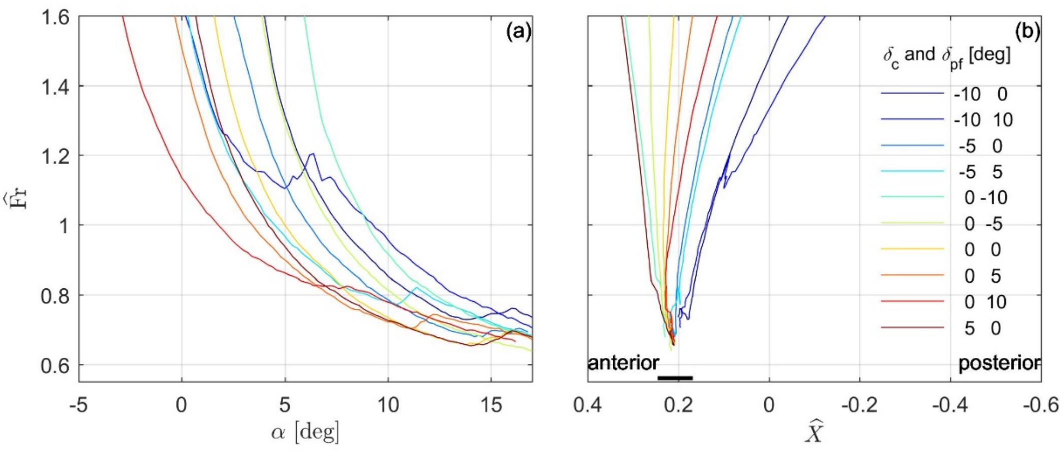

Figure S6: The scaled speed (a) and the scaled distance between the centers of mass and buoyancy (b) that balance the shark in a free (unpowered) glide. This figure is based on the data shown on Fig. S4.

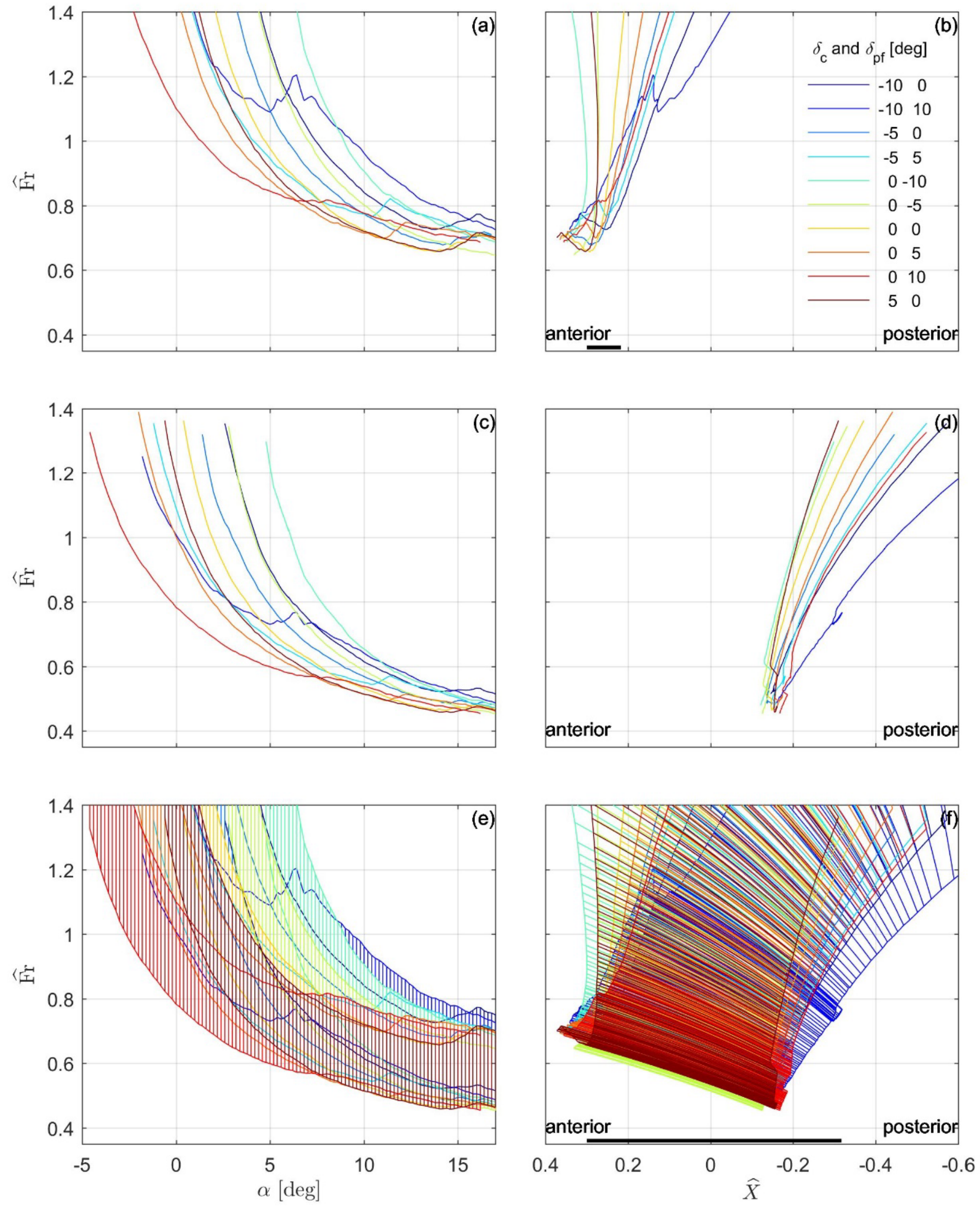

Figure S7: Same as Fig. S5 but in ascent with $\gamma = 20°$. Note that the vertical scales have been shifted down by 0.2 as compared to Fig. S5.
